# Moderating Effect of eHealth Literacy on the Associations of Coronaphobia With Loneliness, Irritability, Depression, and Stigma in Chinese Young Adults: Bayesian Structural Equation Model Study

**DOI:** 10.2196/47556

**Published:** 2023-09-29

**Authors:** Richard Huan Xu, Ho Hin Chan, Lushaobo Shi, Ting Li, Dong Wang

**Affiliations:** 1 Department of Rehabilitation Sciences Hong Kong Polytechnic University Kowloon China (Hong Kong); 2 Department of Applied Mathematics Hong Kong Polytechnic University Kowloon China (Hong Kong); 3 School of Health Management Southern Medical University Guangzhou China

**Keywords:** coronaphobia, eHealth literacy, Bayesian statistics, structural equation modeling, mediating effect, mental health

## Abstract

**Background:**

The COVID-19 pandemic has led to an increase in known risk factors for mental health problems. Although medical information available through the internet and smartphones has greatly expanded, people’s ability to seek, eschew, and use reliable web-based medical information and services to promote their mental health remains unknown.

**Objective:**

This study aims to explore the associations between coronaphobia and 4 frequently reported mental health problems, loneliness, irritability, depression, and stigma, during the COVID-19 pandemic and to assess the moderating effects of eHealth literacy (eHL) on the adjustment of these relationships in Chinese young adults.

**Methods:**

The data used in this study were collected from a web-based survey of the general Chinese population, aged between 18 and 30 years, conducted in China between December 2022 and January 2023. A nonprobability snowball sampling method was used for data collection. A Bayesian structural equation model (BSEM) using parameter expansion was used to estimate the moderating effect of eHL on the relationship between coronaphobia and psychological problems. The posterior mean and 95% highest density intervals (HDIs) were estimated.

**Results:**

A total of 4119 participants completed the questionnaire and provided valid responses. Among them, 64.4% (n=2653) were female and 58.7% (n=2417) were rural residents. All measures showed statistically significant but minor-to-moderate associations (correlation coefficients ranged from −0.04 to 0.65). Significant heterogeneity was observed between rural and urban residents at the eHL level, and coronaphobia was observed. The BSEM results demonstrated that eHL was a significant moderator in reducing the negative effects of coronaphobia on loneliness (posterior mean −0.0016, 95% HDI −0.0022 to −0.0011), depression (posterior mean −0.006, 95% HDI −0.0079 to −0.004), stigma (posterior mean −0.0052, 95% HDI −0.0068 to −0.0036), and irritability (posterior mean −0.0037, 95% HDI −0.0052 to −0.0022). The moderating effects of eHL varied across the rural and urban subsamples.

**Conclusions:**

Using BSEM, this study demonstrated that improving eHL can significantly mitigate the negative effects of coronaphobia on 4 COVID-19–related mental health problems in Chinese young adults. Future eHL initiatives should target rural communities to ensure equal access to information and resources that can help protect their mental health during the pandemic.

## Introduction

The COVID-19 pandemic, which originated in China and spread worldwide, has continued its impact for more than 3 years. This has led to an increase in the known risk factors for mental health problems, such as social isolation, loneliness, inactivity, and limited access to health and social care services [1,2]. Studies have indicated that more than half of those interviewed have experienced moderate-to-severe psychological effects caused by fear of COVID-19, and approximately three-quarters have expressed concern about the health of their family members [3-5].

Experiencing strong fear is normal under uncertain circumstances [6]. However, the COVID-19 pandemic has disrupted people’s ability to lead normal lives. Unlike previous flu epidemics, the fear associated with COVID-19 extends beyond the virus itself, impacting mental health in various ways. This excessive fear response to contracting COVID-19 has led to extreme concern over physiological symptoms, significant stress regarding personal and occupational loss, and avoidance of public places and situations [7]. Along with social distancing and strict quarantine policies worldwide, these fears have generated unprecedented stress on individuals, mentally and emotionally. This uncontrolled and persistent fear has resulted in a new and specific type of anxiety called coronaphobia, exacerbating the consequences on mental health [8].

The COVID-19 pandemic has triggered changes in people’s health care–seeking behavior because of restrictions on social contact, highlighting the importance of digital networks and web-based service platforms [9]. The availability of medical information through the internet and smartphones has greatly expanded the sources for people to seek mental health knowledge and web-based services without leaving their homes during the pandemic. Examples include web-based consultations [10], prescriptions [11], and wireless sensors to monitor the medical conditions of patients [12]. However, the quality of web-based information and services can vary in terms of verifiability, bias, and source. To efficiently navigate the internet, possessing good eHealth literacy (eHL) is particularly important. eHL is an essential skill that ensures people can access reliable web-based medical information and care [13]. Studies have shown that eHL can be useful in alleviating health disparities and improving patient experiences. Equipped with advanced eHL, individuals can search, evaluate, and apply massive amounts of information available on the internet, leading to a significant reduction in their psychological stress [14,15].

The relationship between eHL and psychological status has been widely discussed but is limited in the context of the COVID-19 pandemic. Yang et al [16] found that high eHL was significantly associated with lower levels of depression, insomnia, and posttraumatic stress disorder in 15,000 Chinese individuals. Xu et al [17] also discovered that young people with high eHL who use web-based mental health–related information tend to have a more positive attitude toward seeking mental health services and reporting better mental well-being. Another study demonstrated that rural residents with high eHL were more willing to use telemedicine and had better health status, including mental health, at the beginning of the COVID-19 pandemic [18]. The psychological impact of the pandemic has been moderate to severe because of isolation or quarantine measures. Given the rise of the digital era, understanding how people use the internet to manage their mental health during a pandemic is vital.

Research indicates that eHL can help mitigate the negative impact of COVID-19 fear on the quality of life during the pandemic among diverse populations, including patients [19] and university students [20,21]. Higher levels of eHL are associated with better adherence to preventive measures and improved psychological well-being. Additionally, abundant evidence exists regarding the relationship between coronaphobia and mental health during pandemics. Amin [22] demonstrated that coronaphobia persists among health care professionals and results in psychological symptoms that affect mental health. Yayla and İlgin [23] and Labrague and De Los Santos [24] indicated that nurses’ psychological well-being was significantly affected by coronaphobia. Although a recent study found that health literacy benefits the management of coronaphobia [25], the impact of eHL on coronaphobia during the pandemic has not been sufficiently studied. Considering that the pandemic has profoundly altered how people seek, use, and evaluate health care services, the role of eHL in this transformation should receive more attention.

However, alongside the pandemic, numerous rumors, hoaxes, and misinformation regarding the prevalence, outcomes, and prevention of COVID-19 have circulated on social media at an alarming rate [3-5]. These false claims have generated mass hysteria and panic regarding COVID-19, leading to unexpected and enduring psychological problems for the public [26]. Research has demonstrated that unreliable web-based information about the pandemic has led to heightened anxiety and even lethal mental health ramifications [27,28], which are potentially more detrimental in the long term than the virus itself. Despite this, evidence regarding the role of eHL in managing the impact of coronaphobia on psychological problems and mental illnesses during the pandemic is lacking.

This study aims to provide empirical evidence of the moderating effects of eHL on the relationship between coronaphobia and a wide range of mental health problems frequently reported during the pandemic in young adults in China. Exploration in young adults is particularly important because they are highly exposed to smartphones and the internet, which increases the likelihood of experiencing web-related psychological problems. Research has confirmed the profound impact of the pandemic on young adults, disrupting their education, routines, and peer interactions, thereby raising concerns about potential long-term mental health outcomes in this population [29].

## Methods

### Data and Participants

The data used in this study were obtained from a web-based survey conducted in China between December 2022 and January 2023. The study included participants who met the following criteria: (1) participants aged between 18 and 30 years, (2) participants able to read Chinese, and (3) participants able to provide informed consent. The survey was conducted at a medical university in Guangzhou, China. The research team contacted university officers in charge of student affairs and discussed the data collection plan. A link containing the study introduction and questionnaire was sent to all the students through the university’s internal network using WeChat software (Shenzhen Tencent Computer Systems Company Limited), a multipurpose messaging app. To improve the diversity of the samples, a nonprobability snowball sampling method was used for recruitment. This method is commonly used in quantitative studies [30,31]. The research team did not directly recruit participants other than appointed university students but encouraged them to contact their peers and invited them to join the survey through their personal social networks. To maintain the momentum of the snowball sampling, the research team collaborated with university officials and sent 4 reminders to all students, encouraging them to further disseminate the survey link.

The questionnaire was created using the web-based platform of a professional surveying company (Wenjuanxing), with an informed consent statement appended to it. Interested participants accessed the questionnaire by clicking on the provided link. The first page of the questionnaire focused on obtaining informed consent. Participants had to read, scroll down to the end, click the agree button, and electronically sign it before proceeding to the actual survey. Once participants confirmed their participation, they were presented with a structured questionnaire containing questions about their demographics, socioeconomic status, mental health status, and eHL. Participants required approximately 15 minutes to complete the entire questionnaire.

### Measures

#### eHealth Literacy

The eHealth Literacy Scale (eHEALS) was used to measure participants’ knowledge and perceived skills in finding, evaluating, and applying eHealth information to manage health problems [32]. It was developed based on a framework comprising 6 dimensions to understand and use eHealth information. The eHEALS has 8 items (eg, I know how to find helpful health resources on the internet) rated on a 5-point Likert scale. The total eHEALS scores range from 8 to 40. Higher scores indicate greater perceived eHL. Its psychometric properties in the Chinese population have been confirmed by Xu et al [33].

#### Coronaphobia

Coronaphobia was assessed using the COVID-19 Phobia Scale (C19P) [34]. This self-report instrument was developed and validated to measure COVID-19 phobia levels. C19P includes 4 factors (psychological, somatic, social, and economic) with 20 items (eg, the fear of coming down with COVID-19 makes me very anxious) rated on a 5-point Likert scale from “strongly disagree” to “strongly agree.” The total C19P score ranges from 20 to 100, with a high score indicating severe coronaphobia. The psychometric properties in the Chinese population have been reported by Chi et al [35].

#### Loneliness

The University of California, Los Angeles (UCLA) 3-item loneliness scale (ULS-3) was used to determine the perception and degree of loneliness [36]. It comprises 3 items (eg, how often do you feel that you lack companionship?) to assess an individual’s feelings of companionship, being left out, and being isolated. Each item is rated on a 3-point Likert scale. The total ULS-3 score ranges from 3 to 9, with a higher score indicating a higher level of perceived loneliness. The psychometric properties of the Chinese ULS-3 have been reported by Liu et al [37].

#### Irritability

The brief irritability test (BIT) developed by Holtzman et al [38] was applied to assess irritation. This brief self-report scale for irritability demonstrates strong reliability and validity in both healthy and clinical populations. It includes 5 items (eg, I have been feeling like I might snap) rated on a 6-point Likert scale (1 to 6). A higher score indicates a higher level of irritation. In this study, the research team translated the BIT from English to Chinese using a standard process (2 forward and 2 backward translations). The Chinese version of the BIT is presented in Multimedia Appendix 1. In this study, the Cronbach α for the Chinese BIT was .89 indicating good internal consistency. Confirmatory factor analysis supported a one-factor structure (comparative fit index=0.99 and root mean square error of approximation=0.03).

#### Depression

Depressive symptoms were assessed using the Patient Health Questionnaire-9 (PHQ-9). The PHQ-9 is one of the most widely used instruments for screening, diagnosing, monitoring, and measuring depression severity. It consists of 9 items (eg, little interest or pleasure in doing things) rated on a 4-point Likert scale (0-3). A total score between 5 and 9 indicates mild depression, 10 and 14 indicates moderate depression, and 15 and above indicates moderately severe depressive symptoms. The psychometric properties of the Chinese version of the PHQ-9 have been reported in different populations [39].

#### Anticipated Stigma

COVID-19–related stigma was measured using the revised Chronic Illness Anticipated Stigma Scale (r-CIASS) [40]. It has 6 items (eg, [if I was to get coronavirus...] a friend or family member would be angry with me), reflecting how individuals would be treated if they were to become infected with COVID-19, which are rated on a scale from very unlikely (1) to very likely (4). Higher scores indicate a higher degree of stigma. In this study, the research team translated the r-CIASS from English to Chinese using a standard process (2 forward and 2 backward translations). The Chinese version of the r-CIASS is presented in Multimedia Appendix 2. In this study, Cronbach α for the Chinese r-CIASS was .92. Confirmatory factor analysis supported a one-factor structure (comparative fit index=0.99 and root mean square error of approximation=0.04).

### Statistical Analysis

A descriptive analysis was conducted to examine the participants’ background characteristics and mental health statuses. The min-max normalization method was applied to perform a linear transformation of the original data to then scale the data in the range between 0 and 100.

To assess the moderating effect of eHL on the relationship between coronaphobia and the 4 psychological problems, the Bayesian structural equation model (BSEM) via parameter expansion was used [41].

**Figure 1:**
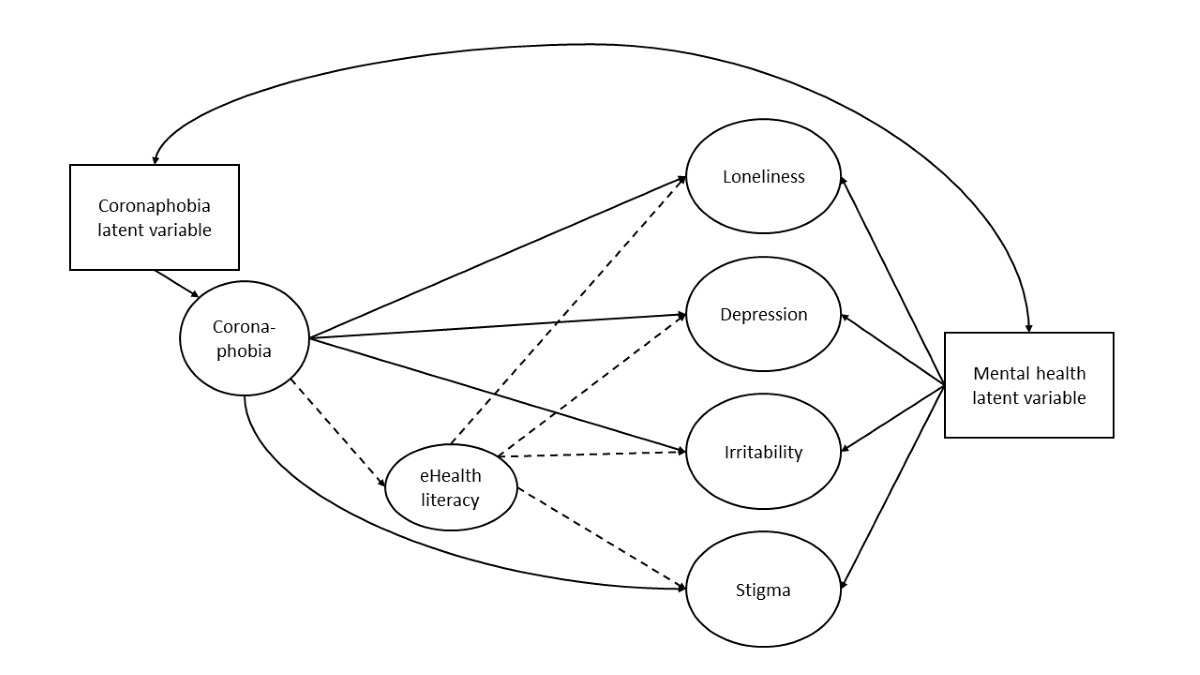
Conceptual framework of Bayesian structural equation model analysis.

Bayesian methods are becoming increasingly popular in various fields because they enable the simultaneous estimation of all cross-loadings and residual correlations in a particular model, a task that is not possible with a maximum-likelihood (ML)–based estimation. Traditionally, ML estimation considers parameters as constants and aims to identify the best-fitting models for certain parameters using research data. However, Bayesian estimation involves combining data likelihoods and prior distributions to construct a posterior distribution. This combination has led researchers to apply the Bayes theorem to the estimation processes [42]. Using BSEM can improve the estimation of parameter and latent variables, enable statistics for model comparison, and provide more reliable results for smaller samples [43]. In this study, the BSEM incorporates 2 latent variables: one representing coronaphobia and the other encompassing loneliness, depression, stigma, and irritation. These 2 latent variables exhibit correlated residuals. Figure 1 illustrates the conceptual framework of the BSEM.

Given the reparameterization of the covariances, the prior distributions on the working model parameters were specified. A subset of 800 participants was randomly selected from the full data set to calculate the prior probability. Their demographics are presented in Multimedia Appendix 3. For the BSEM, we ran 2 chains, each with 21,000 burn-in iterations and 4000 inference iterations. We verified the convergence of the trace plots for all variables and reported the statistics of the latent and regression variables. Information about the posteriors was summarized in terms of the mean and SD, and a 95% highest density interval (HDI) of the posterior mean was calculated. We focused on the direct effects of the 4 aforementioned relationships involving the coronaphobia variable. The 4 indirect effects were calculated using Sobel test [44]. To calculate the indirect effect of the eHL variable on the direct effect of coronaphobia on loneliness, we denote the coefficients of coronaphobia for eHL and eHL for loneliness as *c_Coronaphobia,eHL_* and *c_eHL,loneliness_*, respectively. Their SDs are denoted as SD*c_Coronaphobia,eHL_* and SD*c_eHL,loneliness_*, respectively. Using the following formula derived from the Sobel test, we calculate the posterior mean *c_indirect(Coronaphobia,eHL,loneliness)_* and SD*c_indirect(Coronaphobia,eHL,loneliness)_* of the indirect effect. The same method was used to calculate the posterior mean and SD of the indirect effects of the other 3 variables. R software was used for all statistical analyses. The significance level was set as <.05.

*c_indirect(Coronaphobia,eHL,loneliness)_*=*c_Coronaphobia,eHL_*⋅*c_eHL,loneliness_***(1)**

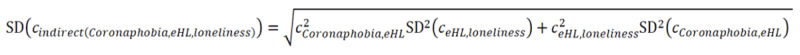

**(2)**


### Ethics Approval

The study protocol and informed consent were approved by the Human Research Ethics Committee of Hong Kong Polytechnic University (Ref No.: HSEARS-20210328002). Written informed consent was obtained from all the participants. All study data were collected anonymously and no compensation was provided to any participant.

## Results

### Participants’ Characteristics

Table 1 shows that 4119 participants joined the survey and completed the questionnaire (completion rate=94.9%). Among them, 64.4% (n=2653) were female, 58.7% (n=2417) were rural residents, and nearly one-third reported a perceived family income lower than the local average (n=1220). Regarding COVID-19–related characteristics, approximately 70% (n=3024) had been infected with COVID-19. Approximately 86.6% (n=2567) and 90.6% (n=3733) reported having experienced a lockdown and working or studying from home because of COVID-19, respectively.

**Table 1:** Background characteristics of participants (n=4119).

Characteristics		Values, n (%)
**Gender**		
	Male	1466 (35.6)
	Female	2653 (64.4)
**Age**		
	22 years or younger	2120 (51.5)
	Older than 22 years	1999 (48.5)
**Educational attainment**		
	Undergraduate or below	3115 (75.6)
	Postgraduate or above	1004 (24.4)
**Residence registry**		
	Rural resident	2417 (58.7)
	Urban resident	1702 (41.3)
**Perceived family income**		
	Lower than average	1220 (29.6)
	Equal to average	2601 (63.1)
	Higher than average	298 (7.2)
**People infected with COVID-19**		
	No	1175 (30)
	Yes	3024 (70)

### Profiles of and Correlations Between Measures

In terms of eHL, the participants reported a mean eHEALS score of 28.6 (8-40). After normalization, the participants demonstrated a slightly higher level of phobia (C19P’s mean 44; IQR 0-100) than other types of mental health problems (mean 24-40.2; IQR 0-100). The correlation analysis revealed a statistically significant but minor-to-moderate association between the measures (Table 2).

**Table 2:** Profiles of measures and the correlations between them.

		Mean (SD)	Median (IQR)	Normalized mean (0-100)	1	2	3	4	5
**1. eHEALS^a^**		28.6 (6.1)	29 (8-40)	64.5					
	Correlation, *r*				—^b^				
	*P* value				—				
**2. C19P^c^**		55.2 (16.6)	54 (20-100)	44					
	Correlation, *r*				–0.14	—			
	*P* value				<.001	—			
**3. ULS-3^d^**		5 (1.6)	5 (3-9)	33.4					
	Correlation, *r*				–0.08	0.29	—		
	*P* value				<.001	<.001	—		
**4. BIT^e^**		15.1 (4.9)	15 (5-30)	40.2					
	Correlation, *r*				–0.04	0.36	0.5	—	
	*P* value				.01	<.001	<.001	—	
**5. PHQ-9^f^**		15.5 (5.8)	14 (9-36)	24					
	Correlation, *r*				–0.07	0.37	0.54	0.65	—
	*P* value				<.001	<.001	<.001	<.001	—
**6. r-CIASS^g^**		12.1 (4.6)	12 (6-24)	33.7					
	Correlation, *r*				–0.09	0.32	0.42	0.44	0.51
	*P* value				<.001	<.001	<.001	<.001	<.001

^a^eHEALS: eHealth Literacy Scale.

^b^Not available.

^c^C19P: COVID-19 Phobia Scale.

^d^ULS-3: University of California, Los Angeles 3-Item Loneliness Scale.

^e^BIT: Brief Irritability Test.

^f^PHQ-9: Patient Health Questionnaire-9.

^g^r-CIASS: Revised Chronic Illness Anticipated Stigma Scale.

### Coronaphobia and eHL in Different Socioeconomic and COVID-19–Related Status Groups

**Figure 2:**
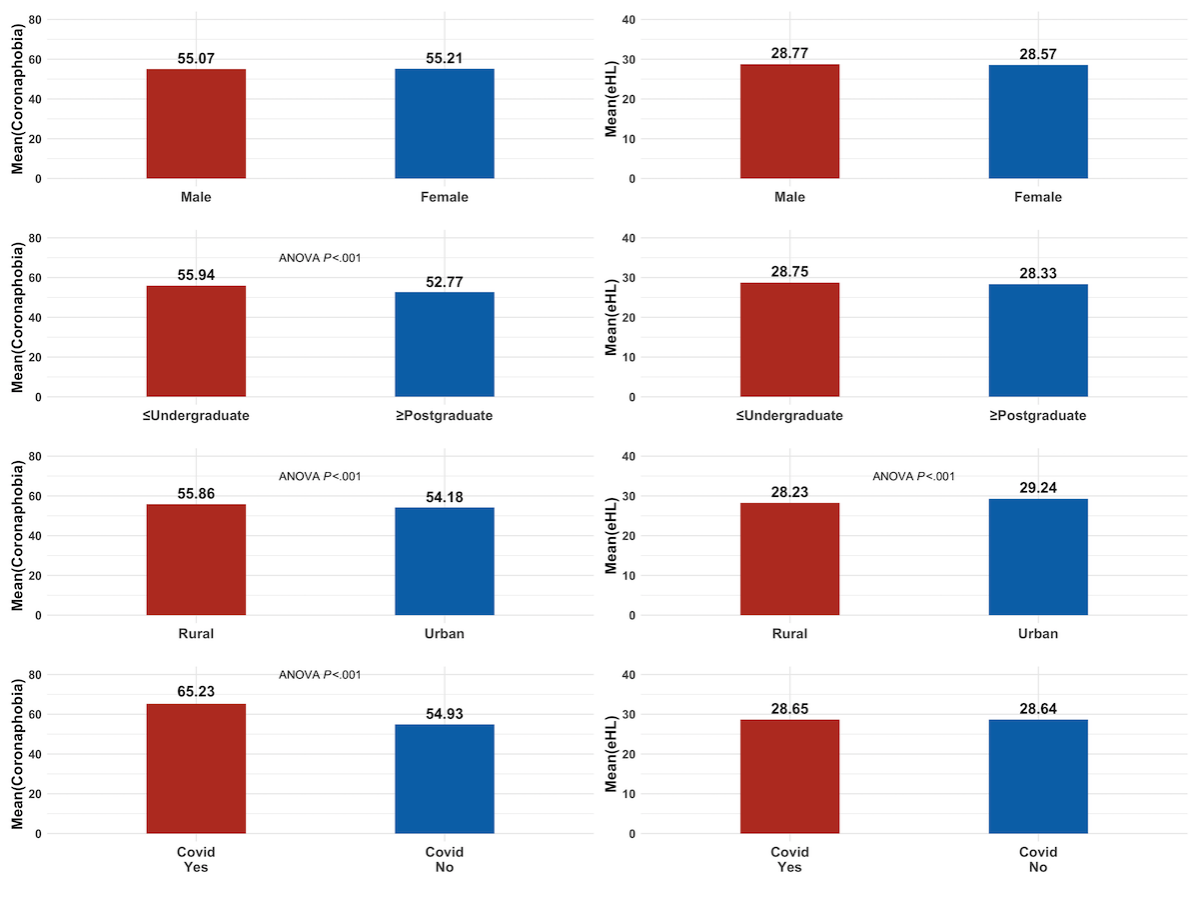
Mean scores of COVID-19 Phobia Scale (C19P) and eHealth Literacy Scale (eHEALS) in subgroups. eHL: eHealth literacy.

Rural residents reported a higher level of coronaphobia but a lower level of eHL than their urban counterparts. Participants with a higher perceived family income tended to have a high level of eHL, but a low level of coronaphobia. Additionally, participants with COVID-19 demonstrated high levels of coronaphobia (Figure 2).

### Direct Effect and the Moderating Effect of eHL

Using the Bayesian approach, we report the results in terms of the posterior distribution. Table 3 shows a significantly strong and positive effect of latent variable 2 on depression, stigmatization, and irritation (with longlines set as the reference). This suggests the presence of a latent variable linked to these 4 variables. Considering the significant difference in eHL and coronaphobia between rural and urban residents, separate analyses were conducted for these 2 subsamples.

**Figure 3:**
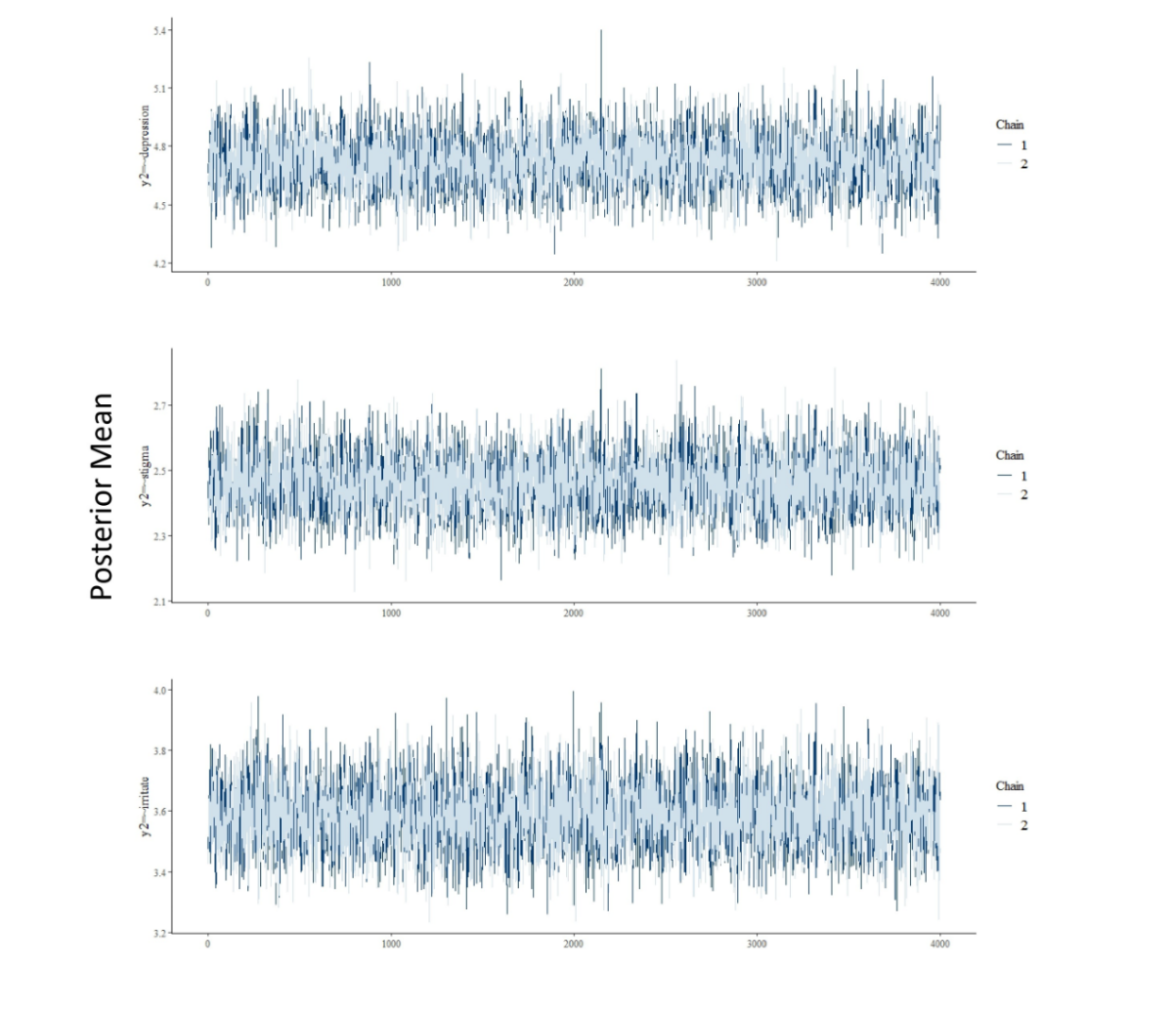
Traceplots of convergence for some model parameters.

The posterior mean and posterior SD of the direct effects of coronaphobia on the 4 psychological problems are presented in Table 4. The coefficients are all statistically significant, as indicated by the 95% HDI. The direct effect of coronaphobia on depression was stronger than that on the other 3 psychological problems. Compared to rural residents, coronaphobia was more likely to lead to a higher level of depression and loneliness but lower levels of stigma and irritability among urban residents. Table 5 shows that eHL was a significant moderator in reducing the negative effect of coronaphobia on loneliness (posterior mean −0.0016, 95% HDI −0.0022 to −0.0011), depression (posterior mean −0.006, 95% HDI −0.0079 to −0.004), stigma (posterior mean −0.0052, 95% HDI −0.0068 to −0.0036), and irritability (posterior mean −0.0037, 95% HDI −0.0052 to −0.0022). The trace plot shows a random scatter around the mean value. Thus, our model results suggest that the chains mixed well and that the model converged (Figure 3). Other trace plots are presented in Multimedia Appendix 4.

**Table 3:** Latent variable coefficients within mental health latent variable using Bayesian structural equation models (BSEMs) and stratified by respondent’s residence registry.

			Posterior, mean (SD)		95% highest density interval
**Full data (n=4199)**					
	Latent→Loneliness	1.000^a^		—^b^	
	Latent→Depression	4.718 (0.142)		4.447-5.007	
	Latent→Stigma	2.463 (0.090)		2.289-2.646	
	Latent→Irritability	3.588 (0.108)		3.381-3.805	
**Rural residents (n=2074)**					
	Latent→Loneliness	1.000^a^		—	
	Latent→Depression	4.815 (0.194)		4.455-5.209	
	Latent→Stigma	2.583 (0.123)		2.348-2.836	
	Latent→Irritability	3.672 (0.150)		3.393-3.974	
**Urban residents (n=2045)**					
	Latent→Loneliness	1.000^a^		—	
	Latent→Depression	4.623 (0.213)		4.222-5.062	
	Latent→Stigma	2.311 (0.135)		2.058-2.583	
	Latent→Irritability	3.502 (0.164)		3.192-3.838	

^a^SD values are not available.

^b^Not available.

**Table 4:** Direct effects of associations between coronaphobia and mental health outcomes using Bayesian structural equation models (BSEMs) and stratified by respondent’s residency registry.

			Posterior, mean (SD)		95% highest density interval
**Full (n=4119)**					
	Coronaphobia→Loneliness	0.068 (0.026)		0.019-0.119	
	Coroanphobia→Depression	0.313 (0.122)		0.083-0.554	
	Coronaphobia→Stigma	0.186 (0.064)		0.066-0.314	
	Coronaphobia→Irritability	0.245 (0.093)		0.071-0.431	
**Rural residents (n=2074)**					
	Coronaphobia→Loneliness	0.066 (0.025)		0.02-0.117	
	Coroanphobia→Depression	0.316 (0.118)		0.095-0.558	
	Coronaphobia→Stigma	0.194 (0.064)		0.075-0.325	
	Coronaphobia→Irritability	0.260 (0.090)		0.089-0.445	
**Urban residents (n=2045)**					
	Coronaphobia→Loneliness	0.072 (0.026)		0.023-0.125	
	Coroanphobia→Depression	0.318 (0.120)		0.096-0.562	
	Coronaphobia→Stigma	0.185 (0.060)		0.073-0.308	
	Coronaphobia→Irritability	0.238 (0.091)		0.068-0.421	

**Table 5:** Moderating effects of eHL^a^ in adjusting associations between coronaphobia and mental health outcomes using Bayesian structural equation models (BSEMs) and stratified by residence registry.

			Posterior, mean (SD)		95% highest density interval
**Full (n=4119)**					
	Coronaphobia→eHL→Loneliness	–0.0016 (0.0003)		–0.0022 to –0.0011	
	Coronaphobia→eHL→Depression	–0.0060 (0.0010)		–0.0079 to –0.004	
	Coronaphobia→eHL→Stigma	–0.0052 (0.0008)		–0.0068 to –0.0036	
	Coronaphobia→eHL→Irritability	–0.0037 (0.0008)		–0.0052 to –0.0022	
**Rural residents (n=2074)**					
	Coronaphobia→eHL→Loneliness	–0.0020 (0.0005)		–0.0029 to –0.0012	
	Coronaphobia→eHL→Depression	–0.0081 (0.0017)		–0.0113 to –0.0048	
	Coronaphobia→eHL→Stigma	–0.0067 (0.0014)		–0.0094 to –0.004	
	Coronaphobia→eHL→Irritability	–0.0060 (0.0014)		–0.0074 to –0.005	
**Urban residents (n=2045)**					
	Coronaphobia→eHL→Loneliness	–0.0013 (0.0003)		–0.0019 to –0.0006	
	Coronaphobia→eHL→Depression	–0.0042 (0.0011)		–0.0064 to –0.0021	
	Coronaphobia→eHL→Stigma	–0.0039 (0.0010)		–0.0059 to –0.002	
	Coronaphobia→eHL→Irritability	–0.0021 (0.0008)		–0.0037 to –0.0006	

^a^eHL: eHealth literacy.

## Discussion

### Principal Findings

The results of the BSEM showed that Chinese young adults with high eHL levels were less likely to report a negative impact of coronaphobia on their mental health. While previous studies have indicated that digital tools and mobile technologies played a vital role in helping people manage their mental health during the pandemic, our study adds to the knowledge that the effectiveness and efficiency of web-based interventions or programs may depend on users’ eHL. This study provides empirical evidence that simply offering web-based mental health services may not be sufficient to enhance the mental health outcomes of young adults; thus, improving their eHL is essential. For policy makers, comprehending and developing an eHealth strategy that creates an interactive relationship between mental health providers, partners, and users and empowers them to use web-based resources to reduce the effect of coronaphobia on their mental health is critical.

Research has demonstrated that the fear of COVID-19 can reduce the use of health care services. For example, one study found that COVID-19–related stigma can cause people to underreport symptoms and avoid health facilities [45]. Moreover, the fear of COVID-19 can cause people to delay or avoid seeking medical attention for non–COVID-19–related illnesses, which can lead to negative health outcomes [46]. Fear can also affect mental health and lead to poor outcomes. Although eHL interventions can help reduce fear and anxiety related to COVID-19, low eHL affects a large percentage of the global population. Naeem and Boulos [47] indicated that low eHL directly contributes to the spread of COVID-19-related web-based misinformation. Lee et al [48] found that 68% of adults were exposed to COVID-19–related misinformation through social networking services or instant messaging. The widespread dissemination of misinformation through social media can render distinguishing between accurate information and falsehoods difficult for people, which can further increase anxiety and fear regarding COVID-19.

Our study identified that people’s ability to find and use health information from digital sources is essential, given the increasing number of people turning to the internet for health information to improve their coping skills against mental health stressors. Participants with high eHL were more likely to find accurate and trustworthy information and resources that could significantly reduce the negative impact of coronaphobia on their mental health. By improving eHL, individuals can better manage their COVID-19–related fear and anxiety and prevent them from developing into a phobia [49]. This can have positive effects on their overall mental health and well-being and complement the efforts to prevent the spread of the virus. Improving eHL can be an important step in this process as it can help people access accurate information and resources that can help them manage their fear and anxiety related to COVID-19.

In China, the COVID-19 pandemic has impacted both urban and rural residents, although not necessarily equally [50]. Our study revealed that rural residents reported significantly higher levels of coronaphobia than urban residents. This finding partially aligns with previous findings that rural residents have been disproportionately affected by the COVID-19 pandemic [50]. Empirical evidence confirms that the expansion of telehealth services is a potential solution for bridging the gap in health care access between rural and urban areas. However, our study suggests that compared to urban residents, rural residents showed a lower level of eHL, which may undermine the efficiency of the intervention to improve mental health during the pandemic, resulting in increased disparity and inequality. This finding is consistent with those of previous studies. For example, Rush et al [18] indicated that rural Canadian residents experienced challenges with telemedicine access because of unreliable internet access and found the service impersonal. Witten and Humphry [51] reported that the eHL of rural community residents was insufficient for the proper understanding and use of technology. Initiating public health campaigns targeting rural communities is vital to improve their eHL and ensure adequate resources and support are provided to these areas to mitigate the impact of the pandemic on their mental health.

Our findings confirmed the positive role of eHL in mitigating the negative impact of the pandemic on mental health. However, this conclusion specifically applies to the populations aged between 18 and 30 years. Although this age group comprises a high proportion of internet users in China, with approximately 1.01 billion active users, further research is needed to explore these associations in other age cohorts. Obtaining empirical evidence across different age groups can provide valuable insights for policymaking in future pandemics, as the threat of COVID-19 persists and new public health crises may emerge.

### Strength and Limitations

A significant advantage of our study is the use of BSEM to estimate the associations. BSEM allows for the incorporation of prior information into the model estimation process and provides a more efficient approximation of the model compared to the standard ML approach [52].

However, this study also has certain limitations. The primary limitation is the use of a snowball sampling method for data collection, which has several disadvantages. First, it is a nonprobabilistic technique that relies heavily on existing relationships between respondents, which can lead to insufficient diversity in respondents’ characteristics. Second, the quality of the data received from the respondents depends on their relationship with the introducer. If the introducer has a good relationship with the respondent, the purpose of the survey can be clearly expressed, which can enhance the likelihood of obtaining valid responses. Finally, unlike other sampling methods, the snowball sampling method shifts the recruitment efforts to the respondents themselves, leading to a loss of control for the research team. In this study, given the nature of the web-based survey, we did not record the recruitment process. Thus, this method may not accurately represent the target population, leading to potential problems with the generalizability of our findings. Another limitation is that the data used in this study were collected during the most severe wave of the COVID-19 pandemic in China. Respondents may, in turn, have experienced strong coronaphobia, which may have led to recall bias.

### Conclusions

This study provides evidence that coronaphobia has a significant negative impact on the mental health of Chinese young adults. However, the findings also highlight the potential of improving eHL to mitigate these negative effects. By improving individuals’ ability to access reliable web-based information about the virus and resources for web-based health services, they may feel more confident and in control of their health, thereby reducing their phobia about COVID-19. Moreover, eHL initiatives should specifically target rural communities to ensure equal access to information and resources and protect their mental health during the pandemic.

## Appendix

Multimedia Appendix 1 The Chinese version of the Brief Irritability Test.

Multimedia Appendix 2 The Chinese version of the Revised Chronic Illness Anticipated Stigma Scale.

Multimedia Appendix 3 Demographics of sample of 800 participants.

Multimedia Appendix 4 Traceplots of all associations.

## Abbreviations

BIT: Brief Irritability Test
BSEM: Bayesian structural equation model
C19P: COVID-19 Phobia Scale
eHEALS: eHealth Literacy Scale
eHL: eHealth literacy
HDI: highest density intervals
ML: maximum-likelihood
PHQ-9: Patient Health Questionnaire-9
r-CIASS: Revised Chronic Illness Anticipated Stigma Scale
UCLA: University of California, Los Angeles
ULS-3: UCLA 3-Item Loneliness Scale

## Data Availability

The data sets generated or analyzed during this study are available from the corresponding author on reasonable request. The raw data supporting the conclusions of this paper will be made available upon reasonable request by contacting the corresponding author.
