# Supplementary material for: Moderating Effect of eHealth Literacy on the Associations of Coronaphobia With Loneliness, Irritability, Depression, and Stigma in Chinese Young Adults: Bayesian Structural Equation Model Study
Source: JMIR Public Health Surveill. 2023 Sep 29;9:e47556. doi: 10.2196/47556 (PMC10576235; doi:10.2196/47556)
Supplement: Multimedia Appendix 1 [file publichealth_v9i1e47556_app1.docx]

**新冠肺炎污名化量表** 如果你感染了新冠肺炎，请想一想你周围的人会有多大可能如以下描述般对待你？

|  | **非常**  **不可能** | **不可能** | **有可能** | **非常**  **有可能** |
| --- | --- | --- | --- | --- |
| 1你的朋友或者家人会对你感到愤怒 | 1 | 2 | 3 | 4 |
| 2你的朋友或家人会因为你生病而责备你 | 1 | 2 | 3 | 4 |
| 3你的朋友或家人会觉得你感染新冠肺炎而生病是你自己的错 | 1 | 2 | 3 | 4 |
| 4你的学校会处罚你 | 1 | 2 | 3 | 4 |
| 5你的同学或老师会歧视你 | 1 | 2 | 3 | 4 |
| 6你的同学或老师会因为你自己生病而责备你 | 1 | 2 | 3 | 4 |
