# Supplementary material for: Moderating Effect of eHealth Literacy on the Associations of Coronaphobia With Loneliness, Irritability, Depression, and Stigma in Chinese Young Adults: Bayesian Structural Equation Model Study
Source: JMIR Public Health Surveill. 2023 Sep 29;9:e47556. doi: 10.2196/47556 (PMC10576235; doi:10.2196/47556)
Supplement: Multimedia Appendix 2 [file publichealth_v9i1e47556_app2.docx]

**烦躁易怒量表** 请指出在**过去两周(包括今天)**, 您有多频繁的出现以下的感受或行为？

|  | **从无** | **极少** | **有时** | **经常** | **较多** | **总是** |
| --- | --- | --- | --- | --- | --- | --- |
| 1.我易生氣愛抱怨 | 1 | 2 | 3 | 4 | 5 | 6 |
| 2.我感觉到容易瞌睡 | 1 | 2 | 3 | 4 | 5 | 6 |
| 3.我被他人激怒 | 1 | 2 | 3 | 4 | 5 | 6 |
| 4.我比平时更易被事情困扰 | 1 | 2 | 3 | 4 | 5 | 6 |
| 5.我感到烦躁 | 1 | 2 | 3 | 4 | 5 | 6 |
