# Supplementary material for: Moderating Effect of eHealth Literacy on the Associations of Coronaphobia With Loneliness, Irritability, Depression, and Stigma in Chinese Young Adults: Bayesian Structural Equation Model Study
Source: JMIR Public Health Surveill. 2023 Sep 29;9:e47556. doi: 10.2196/47556 (PMC10576235; doi:10.2196/47556)
Supplement: Multimedia Appendix 3 [file publichealth_v9i1e47556_app3.docx]

Comparisons between subsample and the rest sample.

|  | | Sample | Nonsample | *P* value |
| --- | --- | --- | --- | --- |
| **Rural or urban** | | | | .85 |
|  | Rural | 400 | 1674 |  |
|  | Urban | 400 | 1645 |  |
|  | Total | 800 | 3319 |  |
| **Sex group** | | | | .45 |
|  | Male | 275 | 1191 |  |
|  | Female | 525 | 2128 |  |
|  | Total | 800 | 3319 |  |
| **Age group** | | | | .38 |
|  | 22 years or younger | 400 | 1720 |  |
|  | Older than 22 years | 400 | 1599 |  |
|  | Total | 800 | 3319 |  |
| **Income group** | | | | 0.91^a^ |
|  | Lower than average | 232 | 988 |  |
|  | Equal to average | 509 | 2092 |  |
|  | Higher than average | 59 | 239 |  |
|  | Total | 800 | 3319 |  |
| **Income group** | | | | .04 |
|  | Undergraduate or below | 582 | 2533 |  |
|  | Postgraduate or above | 218 | 786 |  |
|  | Total | 800 | 3319 |  |

^a^Total is reported.
