# Supplementary figures and images for: Moderating Effect of eHealth Literacy on the Associations of Coronaphobia With Loneliness, Irritability, Depression, and Stigma in Chinese Young Adults: Bayesian Structural Equation Model Study
Source: JMIR Public Health Surveill. 2023 Sep 29;9:e47556. doi: 10.2196/47556 (PMC10576235; doi:10.2196/47556)

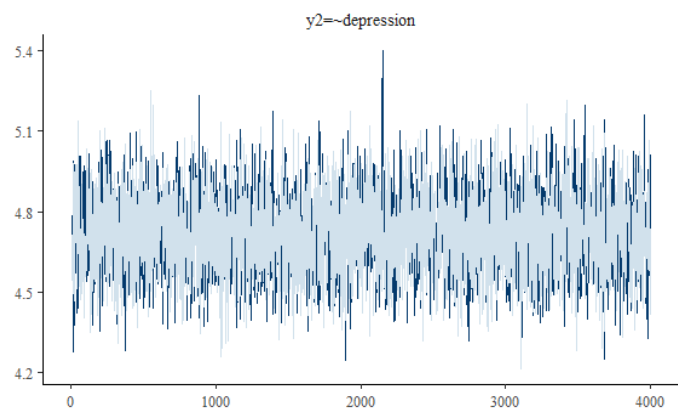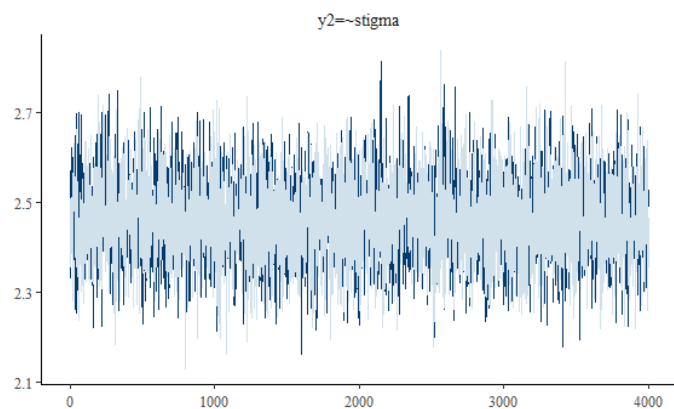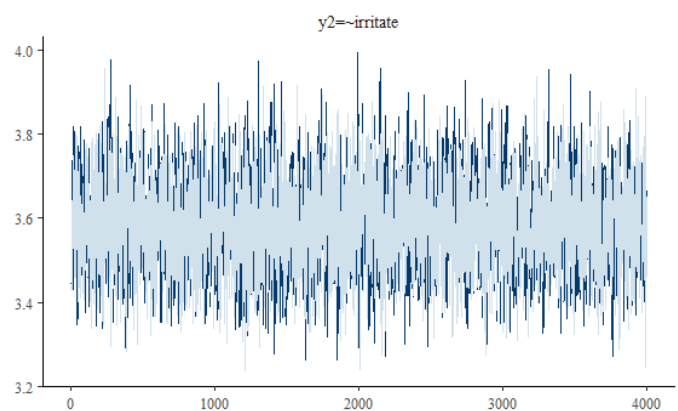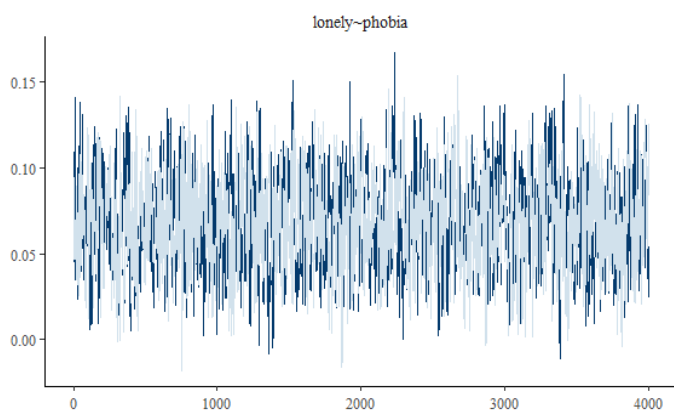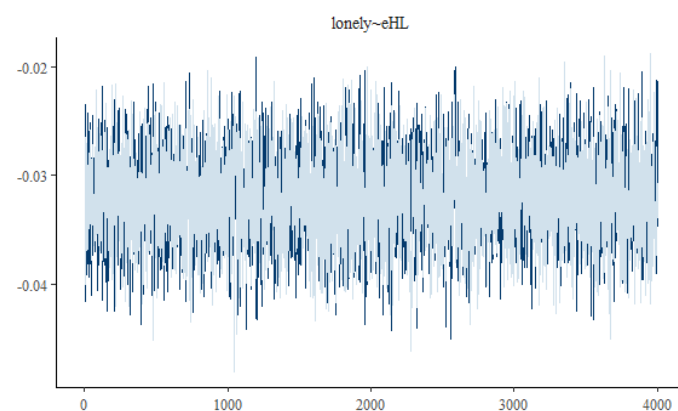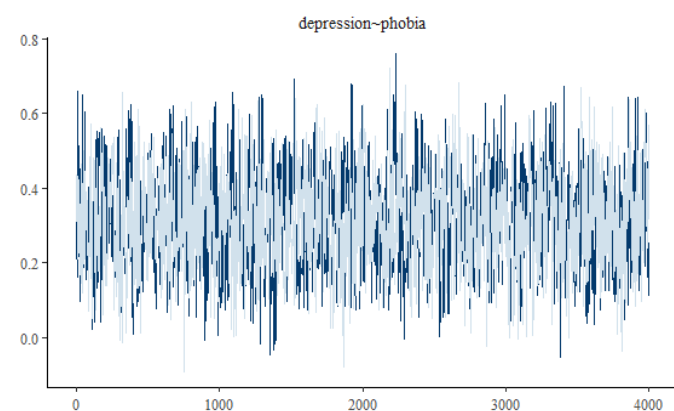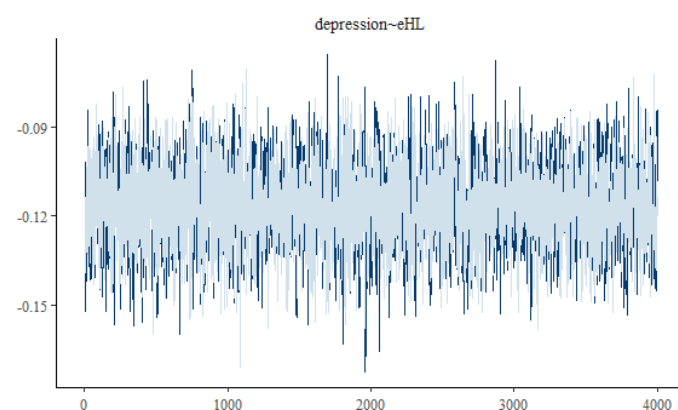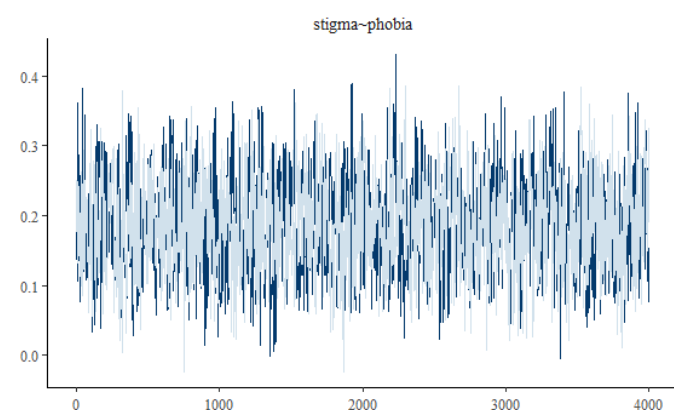

Posterior Mean

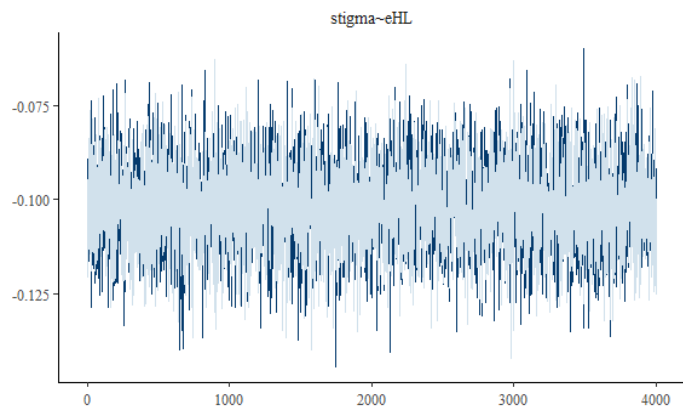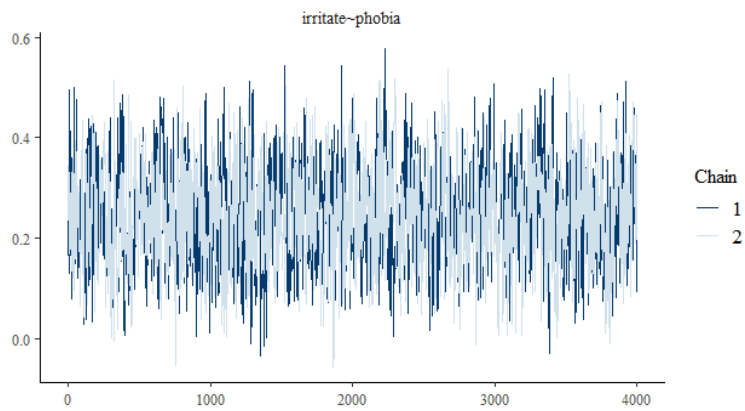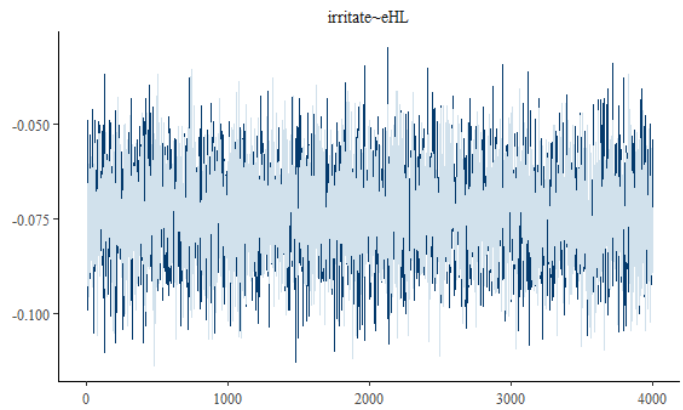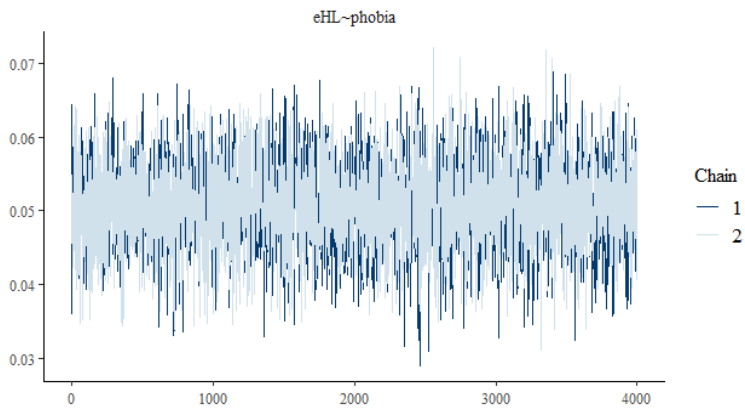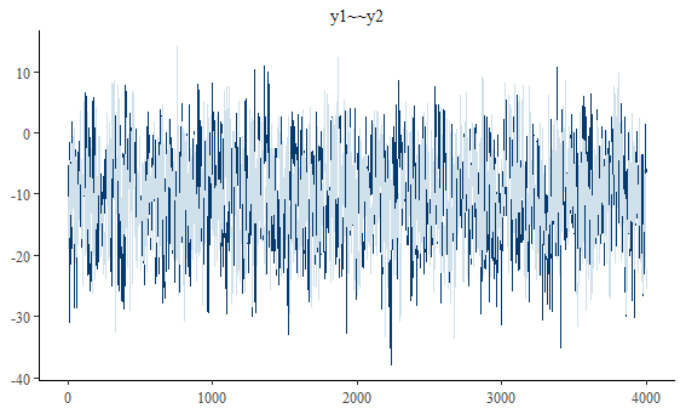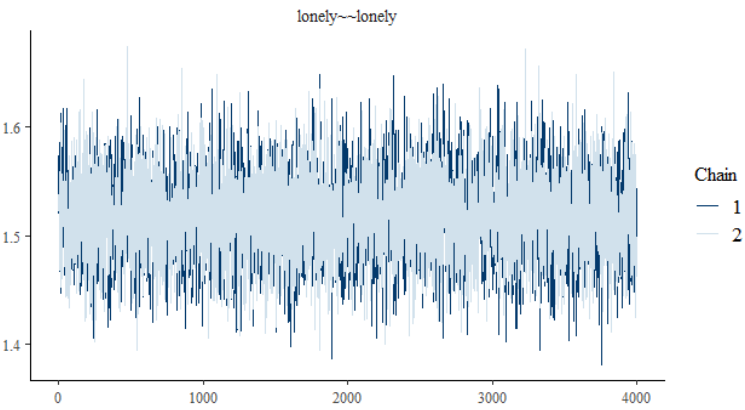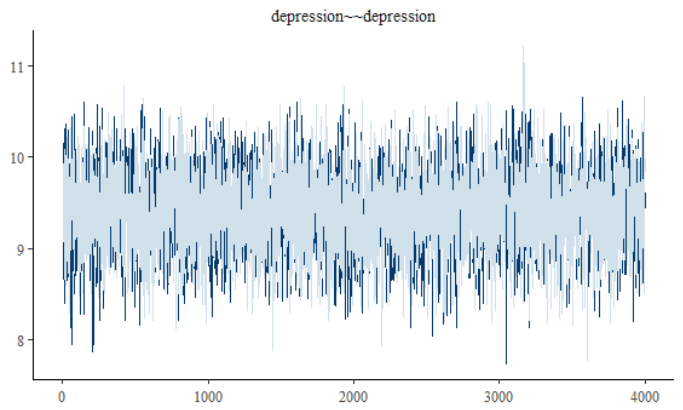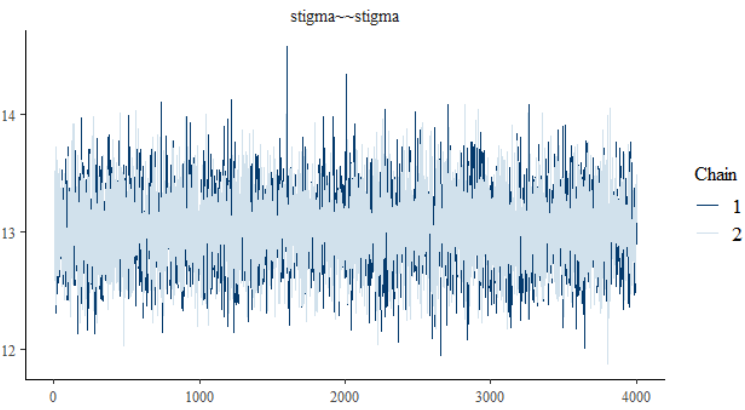

Posterior Mean

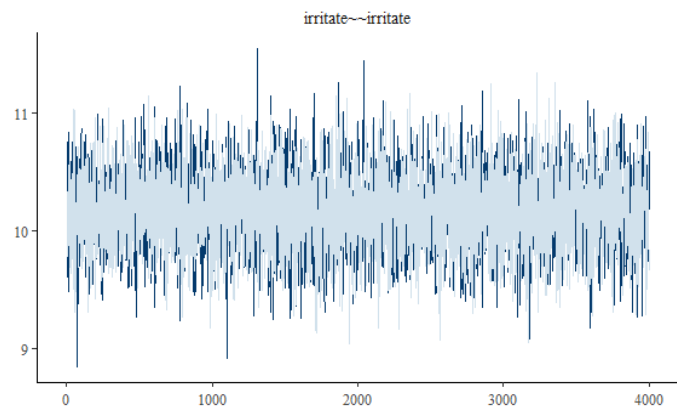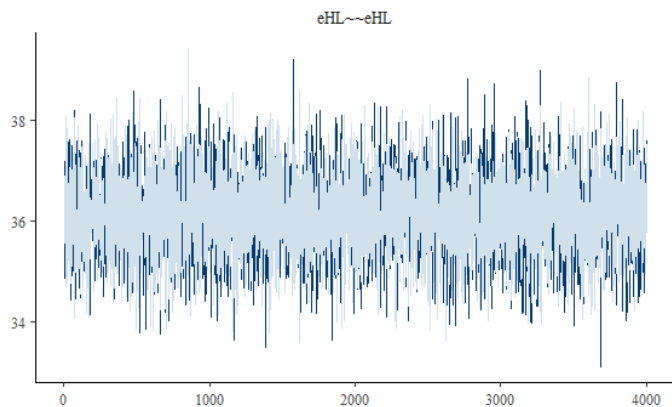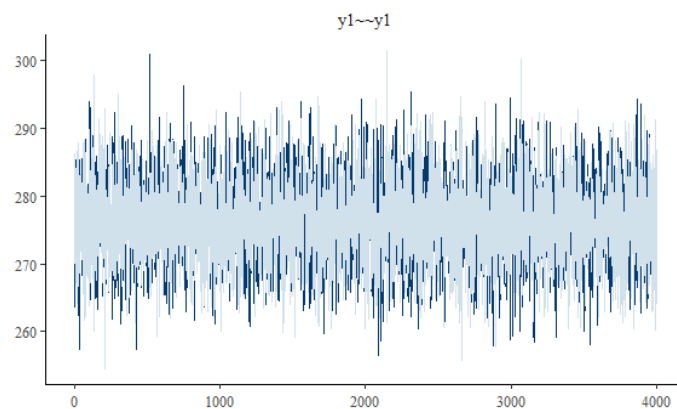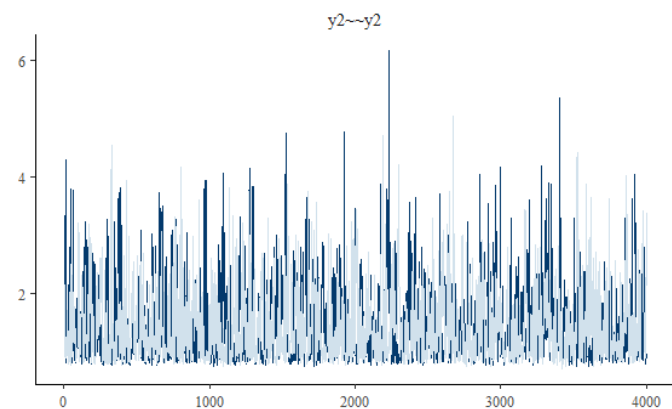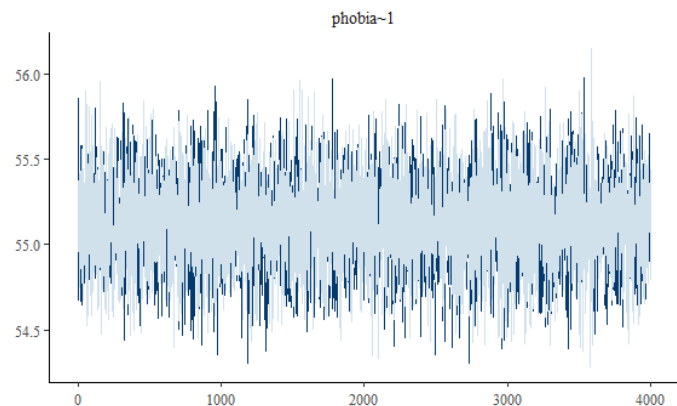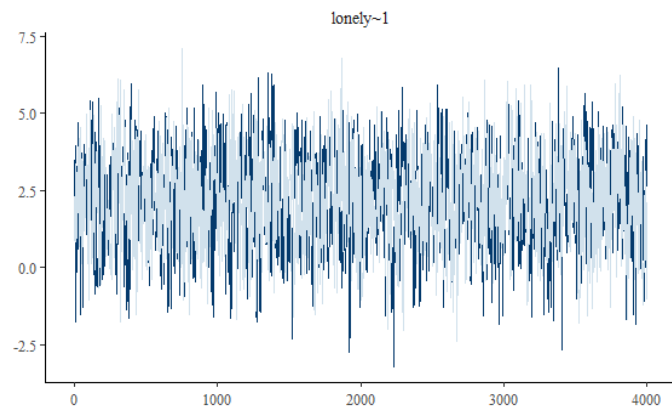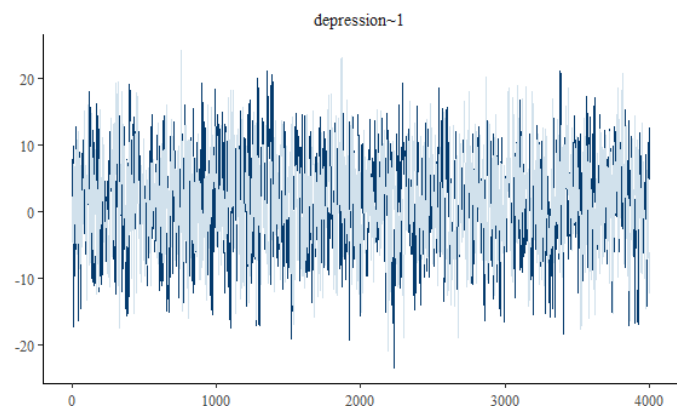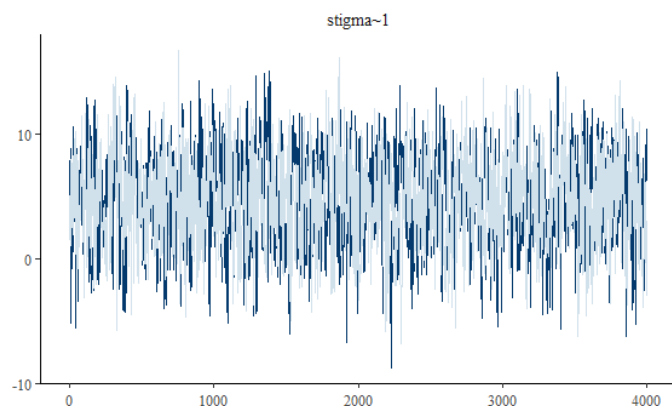

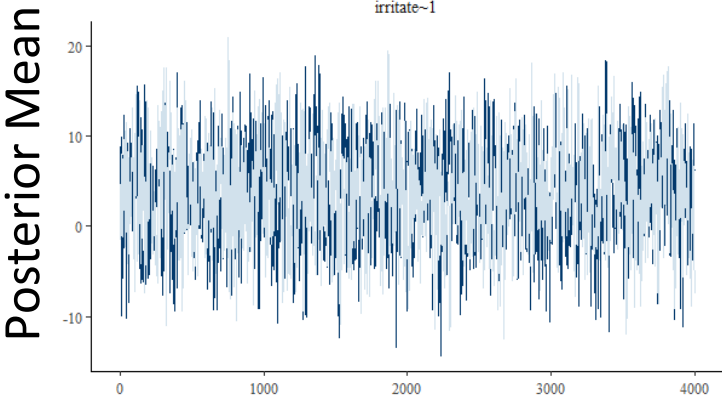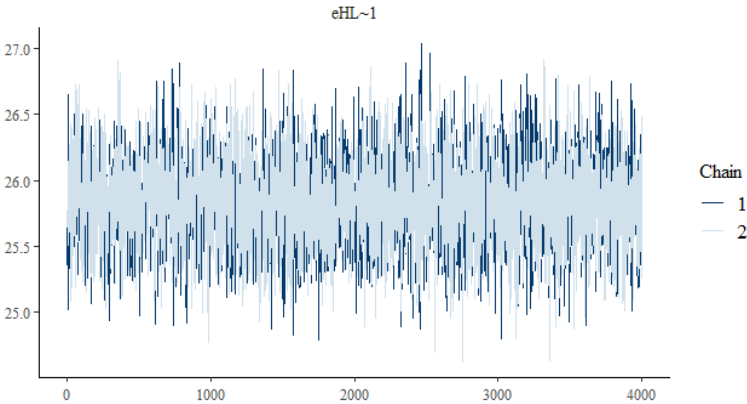

Supplement: Multimedia Appendix 4 [file publichealth_v9i1e47556_app4.pdf]
